# Deep Learning-Enabled Clinically Applicable CT Planbox for Stroke With High Accuracy and Repeatability

**DOI:** 10.3389/fneur.2022.755492

**Published:** 2022-03-11

**Authors:** Yang Wang, Junkai Zhu, Jinli Zhao, Wenyi Li, Xin Zhang, Xiaolin Meng, Taige Chen, Ming Li, Meiping Ye, Renfang Hu, Shidan Dou, Huayin Hao, Xiaofen Zhao, Xiaoming Wu, Wei Hu, Cheng Li, Xiaole Fan, Liyun Jiang, Xiaofan Lu, Fangrong Yan

**Affiliations:** ^1^Department of Radiology, Zhujiang Hospital, Southern Medical University, Guangzhou, China; ^2^State Key Laboratory of Natural Medicines, Research Center of Biostatistics and Computational Pharmacy, China Pharmaceutical University, Nanjing, China; ^3^Department of Radiology, The Affiliated Hospital of Nantong University, Nantong, China; ^4^Department of Endocrinology, Tongren Hospital Affiliated to Shanghai Jiao Tong University School of Medicine, Shanghai, China; ^5^Department of Neurology, Drum Tower Hospital, Medical School and The State Key Laboratory of Pharmaceutical Biotechnology, Institute of Brain Science, Nanjing University, Nanjing, China; ^6^Research & Advanced Algorithm Department of HSW BU, Shanghai United Imaging Healthcare Co., Ltd., Shanghai, China; ^7^Medical School of Nanjing University, Nanjing, China; ^8^Calibration Physical Algorithm Department of CT BU, Shanghai United Imaging Healthcare Co., Ltd., Shanghai, China; ^9^Clinical Workflow and Clinical Verification Department of CT BU, Shanghai United Imaging Healthcare Co., Ltd., Shanghai, China; ^10^Department of CT BU, Shanghai United Imaging Healthcare Co., Ltd., Shanghai, China; ^11^Department of Radiology, The Second Affiliated Hospital of Nantong University, Nantong, China

**Keywords:** stroke, deep learning, computed tomography, automatic cranial scanning, accurate and repeatable images

## Abstract

**Background:**

Computed tomography (CT) plays an essential role in classifying stroke, quantifying penumbra size and supporting stroke-relevant radiomics studies. However, it is difficult to acquire standard, accurate and repeatable images during follow-up. Therefore, we invented an intelligent CT to evaluate stroke during the entire follow-up.

**Methods:**

We deployed a region proposal network (RPN) and V-Net to endow traditional CT with intelligence. Specifically, facial detection was accomplished by identifying adjacent jaw positions through training and testing an RPN on 76,382 human faces using a preinstalled 2-dimensional camera; two regions of interest (ROIs) were segmented by V-Net on another training set with 295 subjects, and the moving distance of scanning couch was calculated based on a pre-generated calibration table. Multiple cohorts including 1,124 patients were used for performance validation under three clinical scenarios.

**Results:**

Cranial Automatic Planbox Imaging Towards AmeLiorating neuroscience (CAPITAL)-CT was invented. RPN model had an error distance of 4.46 ± 0.02 pixels with a success rate of 98.7% in the training set and 100% with 2.23 ± 0.10 pixels in the testing set. V-Net-derived segmentation maintained a clinically tolerable distance error, within 3 mm on average, and all lines presented with a tolerable angle error, within 3° on average in all boundaries. Real-time, accurate, and repeatable automatic scanning was accomplished with and a lower radiation exposure dose (all *P* < 0.001).

**Conclusions:**

CAPITAL-CT generated standard and reproducible images that could simplify the work of radiologists, which would be of great help in the follow-up of stroke patients and in multifield research in neuroscience.

## Introduction

Stroke is a disabling disease, accounting for tens of million deaths during the twenty-first century (1) and computed tomography (CT) plays an indispensable role, as it helps physicians determine the classification of stroke, assess stroke size during follow-up, quantify the penumbra size of ischemic stroke, and support stroke-relevant radiomics studies (2–6). In particular, cranial CT has been heavily used in the examination of children's heads; pertinently, repeated CT examinations during follow-up procedures lead to higher cumulative radiation exposure (7).

Currently, the scope of cranial CT scans basically depends on empirical judgment; such predicaments make it difficult for clinicians to compare and analyze the progress of intracranial lesions. Although emerging intracranial hematoma measurement tools have been developed (8), these volume-based software cannot widely be used in ischemic stroke (9). If the standardization, accuracy and reproducibility of images acquired during follow-up can be ensured, patients (especially children) will be protected due to reduced radiation exposure, and it might be beneficial for neurologists and radiologists to evaluate the condition of stroke patients during follow-up imaging examinations.

The desire to improve the efficacy and efficiency of clinical care continues to drive multiple innovations in practice (10), including artificial intelligence (AI) which has been harnessed in cranial revascularization, aneurysm diagnosis and classification of intracranial hemorrhage (11–14). However, these studies only focused on the diagnosis and evaluation of hematomas, which presented less substantial progress than computer-aided design (CAD) technology. To the best of our knowledge, evidence is scarce regarding the application of AI in assisting cranial imaging; we therefore hypothesize that AI may provide CT with intelligence and allow it to acquire standard, accurate, and reproducible cranial CT images.

To this end, we invented an intelligent CT—Cranial Automatic Planbox Imaging Towards AmeLiorating neuroscience (CAPITAL). It is promising that CAPITAL-CT can be used for follow-up of stroke patients and reduce the additional radiation exposure during imaging examinations.

## Methods And Materials

### Study Design and Data

Prospective validation was applied to 515 newly admitted patients, including 306 admitted by Nanjing Drum Tower Hospital and 209 admitted by other hospitals (Gaochun Dongba Central Hospital [*n* = 96], Kunshan People's Hospital [*n* = 113]). Another 609 retrospective cases were used for independent validation, which means a total of 1,124 cases were enrolled in this study for validation. To be specific, the validation procedure (*n* = 1,124) was divided into three parts: (a) the United Imaging group which was fully evaluated by CAPITAL-CT and included patients from Nanjing Drum Tower Hospital (*n* = 166); (b) the manual but CAPITAL-CT-adjusted group from three hospitals—Nanjing Drum Tower Hospital (*n* = 140), Gaochun Dongba Central Hospital (*n* = 96) and Kunshan People's Hospital (*n* = 113); and (c) the skull CT scanning group in which the scans of the patients from four hospitals—Nanjing Drum Tower Hospital (*n* = 352), Jing Ling Hospital (*n* = 125), The Eighth Affiliated Hospital of Sun Yat-sen University (*n* = 22), and Zhanjiang Hospital Affiliated to Guangdong Medical University (*n* = 110)—were entirely manual. All the CT images collected for this study were generated from scanners produced by multiple manufactories, including GE, Philips, Siemens, Toshiba and United Imaging, with a specific radiation protocol (Supplementary Tables 1, 2). Two radiologists (MPY, 4 years of experience, and YW, 12 years of experience) who were blinded to the information from the automatic measurements performed by CAPITAL-CT were involved in reviewing all the images. To assess CAPITAL-CT performance, an evaluation framework was designed in which both apex and base boundaries were evaluated separately by calculating the distance between the boundary and the gold standard, all patients were clinical patients suspected of having a stroke and were routinely admitted to the hospital and needed to undergo CT scans between February 2018 and December 2020. This study was approved by the Ethics Commissions of the six hospitals, with a waiver of informed consent.

**Figure 1:**
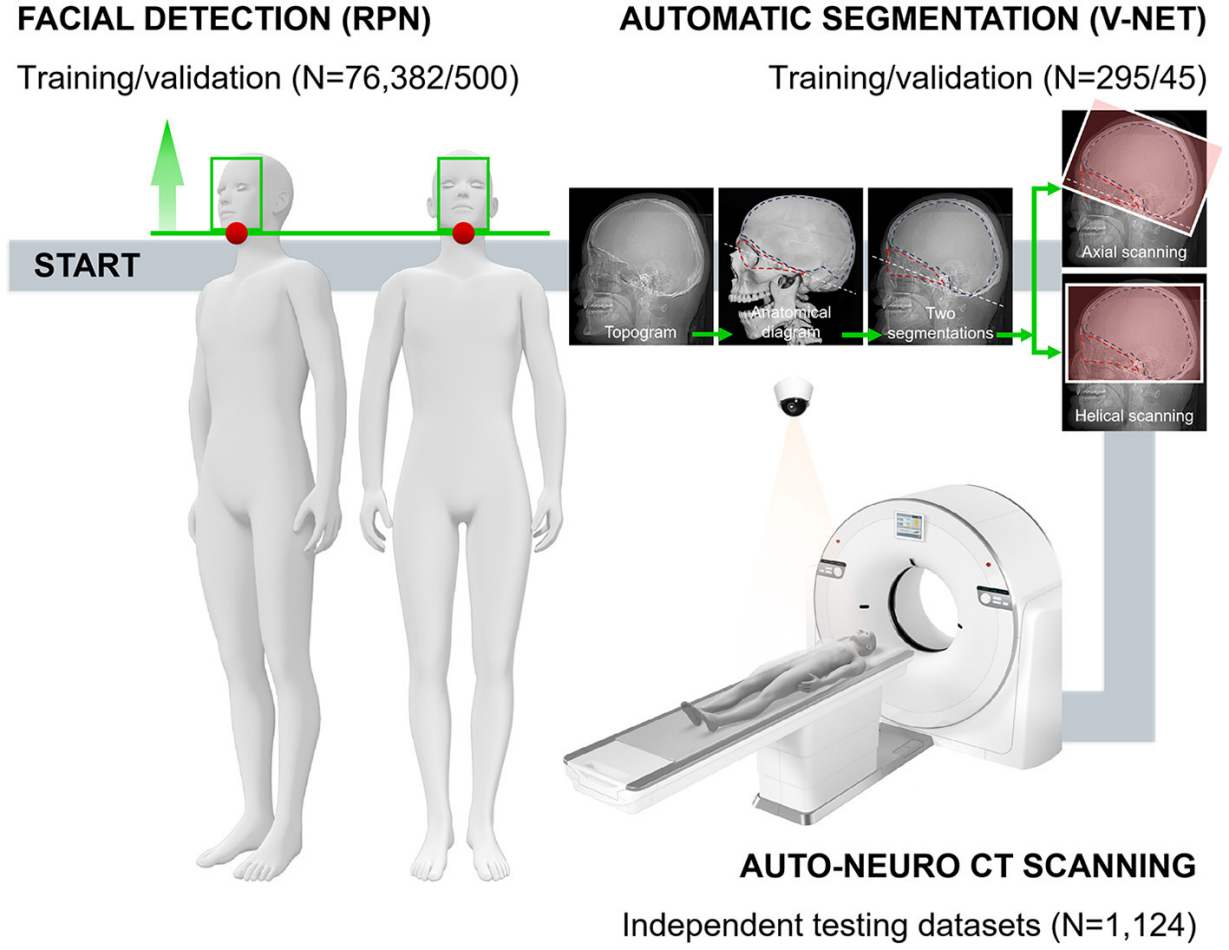
The technical framework of CAPITAL-CT implementation and the entire workflow with respective numbers for each dataset. Following the daily operation logic and habits of imaging to perform the overall process of enriching traditional CT with AI. In the automatic segmentation part, a human skull with dotted lines facilitates readers' understanding of how we chose the segmentation area and the baseline.

The experiment/validation process was divided into three steps (Figure 1 and Supplementary Figure 1): first, the face was detected; second, the two regions of interest (ROIs) were segmented; and third, the scanning planboxes were adjusted according to clinical requirements. To move the subjects to the isocenter automatically, we needed to detect the subjects' face and then calculated a calibration table. More critically, based on the segmentation of two ROIs, the orbitomeatal baseline (OML) was used as the starting point to scan the whole intracranial area to ensure the best scanning quality.

### Image Labeling and Measuring

Four radiologists (CQ, 17 years of experience; XW, 11 years of experience; MPY, 4 years of experience; and YW, 12 years of experience) labeled and measured all images before training. Two ROIs were labeled where ROI1 was the triangular area mainly surrounded by the orbital bone structure and auditory canal; ROI2 was the neurocranium, which is the nearly semicircular area surrounded by the skull (Figure 1). Two radiologists measured key information, including the angle between the scanning baseline and OML, the distance between the left and right anterior horn of the bilateral ventricle, the distance between the left and right anterior horn of the bilateral ventricle along the vertical direction of the brain midline, the number of invalid scanning slices at the top and base (a slice without brain tissue was regarded as invalid) and radiation dose (section Data Availability Statement). These radiologists that reviewed the images were blinded to the information from the automatic measurements performed by CAPITAL-CT.

### Models Development

**Figure 2:**
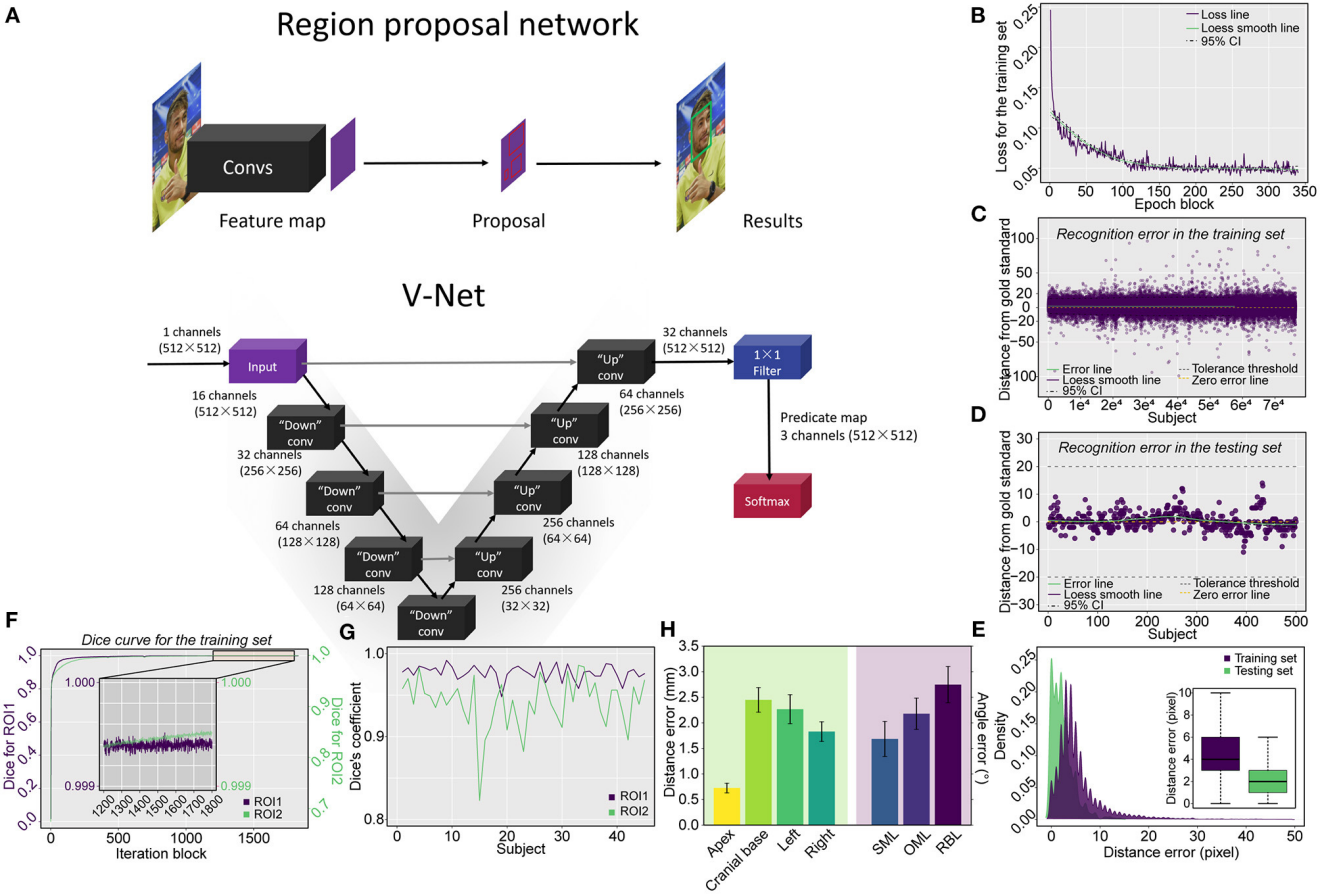
Schematic representation of the applied network architecture, the training process of the RPN and model evaluation *via* the testing set, and the pulmonary segmentation Dice coefficient measurement in both the training and testing procedures. **(A)** The networks include both the RPN used for locating the starting position for the scan and the V-Net used for cranial segmentation. **(B)** The loss curve of the training process converges and trends to zero after 340 epoch blocks (340 × 500 = 170,000 epochs). The errors derived from the training and testing procedures are shown in **(C,D)**, respectively, in which **(E)** the vast majority of errors were located in the tolerance interval of ± 20 mm for the training procedure and all errors were tolerable in the verifying procedure. The Dice coefficient measurement for cranial segmentation in both the training and testing procedures is shown in **(F,G)**. **(F)** With the increase in epoch blocks (1 iteration block equals 231 iterations, a total of 422,499 iterations in the training set including 295 topograms), the Dice coefficient increases rapidly and approaches the peak value of 1. The Dice coefficients of ROI1 (triangular area) and ROI2 (skull) converge and approach 1 in the tail 1,200–1,800 epoch blocks of the training procedure. The exact Dice coefficient curves obtained by deploying the model on the testing set for the triangular area and skull are shown in **(G)**. **(H)** Barplot showing the boundary distance and angle error by deploying the model using the testing set. SML, supraorbitomeatal line; OML, orbitomeatal line; RBL, Reid's baseline.

The region proposal network (RPN) (15) was used for facial detection and to specify the jaw's outer edge as the initial point, colored red, for cranial topogram scans (Figure 2A and Supplementary Data Sharing). V-Net was harnessed to segment the two ROIs on topograms because we reasoned that, compared with U-NET, V-NET has 3-dimensional and multioutput functions (16, 17) where two ROIs in the topograms were labeled by two radiologists as the gold standard. The source code of V-Net was modified to achieve multilabel segmentation on 2-dimensional data according to our previous study (Figure 2A) (18). See technical details experimental software and hardware in Supplementary Methods.

### Camera Installation and Its Calibration Between CT Scanning Couch

**Figure 3:**
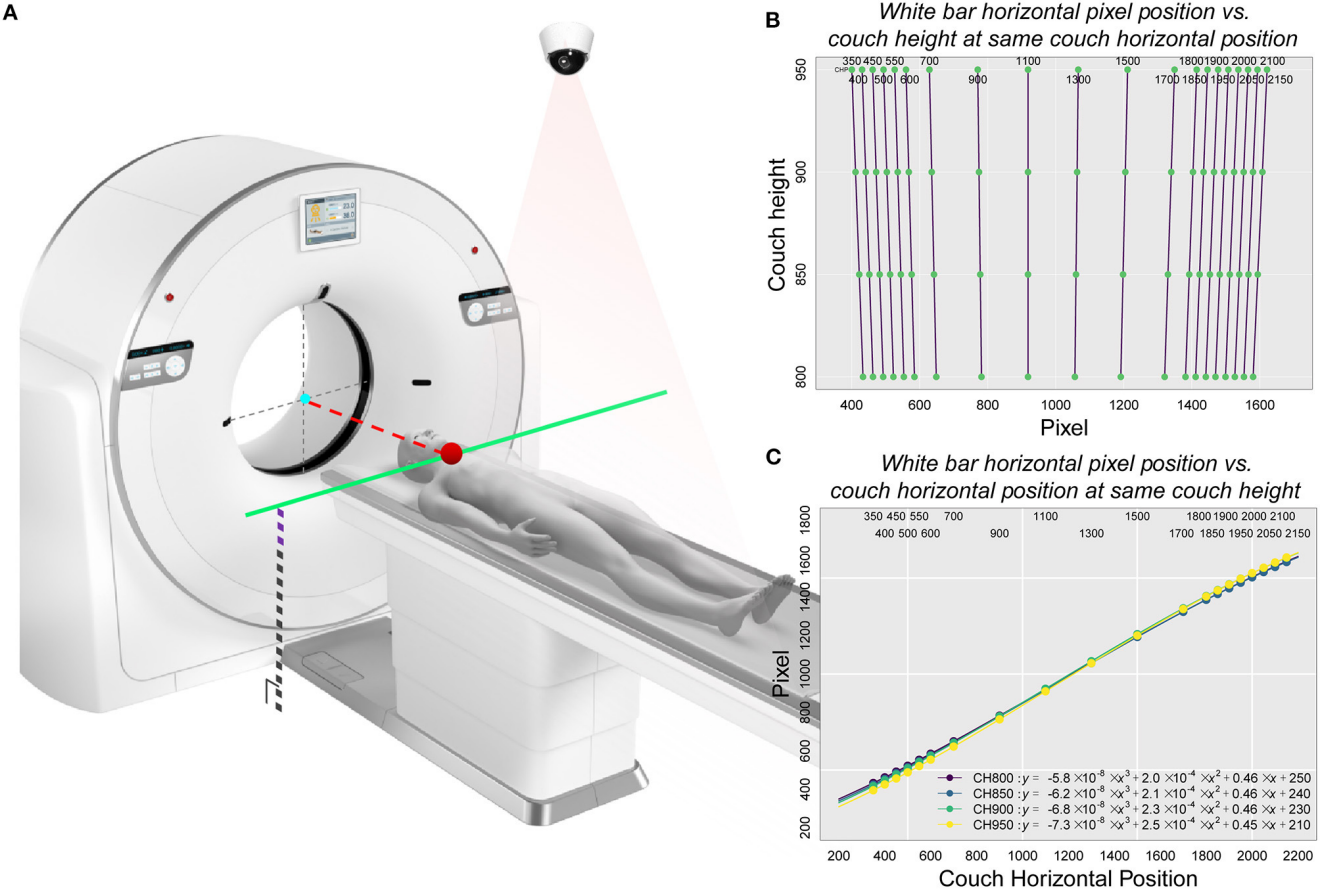
Schematic representation of the principle of calibration used in the study and the corresponding relationship between pixel position and couch height as well as between pixel position and the couch code with the white bar as the correction object. **(A)** The basic principle of automatic positioning of the CAPITAL-CT scanning couch. This diagram included key factors associated with both the CT scanner and the 2-dimensional camera. An important point related to the camera was the pixel position point imaged inside the camera where the key starting position point in the camera detection process was located. This point, which was also a critical part of the scanner, corresponded to the jaw position (the red dot on the solid green line) where the subject was located on the CT scan couch. Since the protocol was already selected, the CT scan couch automatically obtained a fixed couch height and altitude compensation (black dotted lines superimposed with purple dashed lines). Since the position of the isocenter (gray dotted cross with a cyan point) was given, the pixel points of the starting position and the height of the scanning couch were known, the displacement distance of the scanning couch (red dotted line) could be obtained by looking up the calibration table. **(B)** Linear relationship between the horizontal pixel position of the white bar and the couch height under the same couch code. **(C)** Non-linear relationship between the horizontal pixel position of the white bar and the couch code at the same couch height.

According to the camera parameters and the on-site test results, a camera installation range relative to the fixed position of the scanning couch was determined (Supplementary Figures 2, 3). We generated a calibration table as a linkage between the camera and the couch to ensure that the subjects could be automatically moved to the isocenter under the navigation of the camera (Figure 3A). Pattern recognition of the white bar, sparse sampling and fitting were performed to obtain the concise relationship between the couch and the camera pixel (Supplementary Methods).

### Quantification of Accuracy and Repeatability

To quantify the extent of the slices and dose reduction, slices were evaluated by two radiologists and the effective dose per patient was calculated based on the formula mSv = DLP × C_*f*_, where the dose length product (DLP) is a measure of CT tube radiation output/exposure, and C_*f*_ was set as 0.0021 as the conversion factor for cranial CT (19). The absolute mSv value per patient was further scaled as mSv% to eliminate the effect of different scanning protocols selected by different hospitals. Regarding repeatability, the coincidence of the OML at the beginning of the scan, the consistency of the angles, and the symmetry between images within the same level obtained from the first examination and reexamination were measured and compared.

### Statistical Analyses

All statistical analyses were conducted by R4.0.2 using two-sample Student's *t*-test (paired sample *t*-test if appropriate) for continuous data and one-way ANOVA for multiple group comparisons. Continuous variables are summarized as the mean ± standard error of the mean (SEM). A Bland-Altman (BA) plot was used to measure the degree of consistency between two appraisers using the R package “*BlandAltmanLeh*”; differences were compared versus the average of two appraisers using the R package “*smatr*”. A locally estimated scatter plot smoothing (loess) approach was used to generate smooth curves. For all statistical analyses, a two-tailed *P-*value less than 0.05 was considered statistically significant.

## Results

### Facial Detection Performance

The technical skeleton of CAPITAL-CT is delineated in Figure 2A. After 170,000 epochs (500 epochs for each epoch block) of the RPN, the loss curve converged to approximately zero (Figure 2B). We reasoned that an error distance of less than 20 pixels (approximately 5.3 mm) from the gold standard was clinically tolerable and was regarded as a success. The model was first tested on the training set and the error distance ranged from −99 to 96 pixels (4.461 ± 0.018) with a success rate of 98.7% (75,374/76,382; Figure 2C). Furthermore, we verified that the error distance of the testing set that contained 500 human faces ranged from −11 to 14 pixels (2.232 ± 0.101), and the success rate was 100% (Figure 2D). The distance (pixel) from the jaw was also recorded in both training (12,862 images) and testing sets (500 images). The majority of distance errors were located within 10 pixels (approximately 2.7 mm), and the median distance errors were 4 and 2 pixels (approximately 1 and 0.5 mm) for the training and testing sets, respectively, which indicated good facial detection performance (Figure 2E).

### Segmentation Performance

Within 422,499 iterations, the Dice coefficient rapidly increased and tended to be stable and convergent to the peak value of one, regardless of whether evaluating ROI1 or ROI2 (Figure 2F). The Dice coefficient for training ranged from 0.121 to 0.999 for ROI1 and from 1.26 × e^−10^ to 0.999 for ROI2. In the tail 1,200–1,800 iteration blocks, the average Dice coefficient reached a mean value of 0.999 for both ROI1 and ROI2. The final model derived from the training procedure was further deployed on an independent testing set including 45 topograms; we found that the Dice coefficient ranged from 0.948 to 0.992 (0.977 ± 0.001) for ROI1 and 0.823 to 0.986 (0.941 ± 0.005) for ROI2, indicating skull segmentation was accurate due to the average coefficients that were greater than 0.9 (Figure 2G). We further measured the position of the planbox in the testing set (Figure 2H); we found that in all boundaries, the V-Net-derived segmentation kept a clinically tolerable distance error within 3 mm on average. Moreover, we found that a relatively higher error existed in the measurement of the base compared to those of other boundaries (apex: 0.73 ± 0.10; base: 2.45 ± 0.24; left: 2.27 ± 0.28; right: 1.83 ± 0.19), which may be due to the complex anatomy of the cranial base. Additionally, we measured the angle error of three lines, and all lines presented with a tolerable angle error within 3° on average (supraorbitomeatal line [SML]: 1.68 ± 0.34; OML: 2.18 ± 0.31; Reid's baseline [RBL]: 2.75 ± 0.35).

### Calibration Between the Camera and CT Scanning Couch

We decomposed the scanning couch into two parts (Figure 3A), one of which was the relationship between the pixel position and different couch heights under the same couch code. We found that this relationship had almost a perfectly linear fit (*Pixel* = *k* × *Couch Height*+*b*; Figure 3B and Supplementary Tables 3–6). Additionally, we reasoned that under the same couch height, the non-linear relationship between the horizontal pixel position of the white bars and the couch code could be covered by spline interpolation (Figure 3C). When partial data were collected, we used sparse sampling to calculate the corresponding pixel position within the effective operating space range of the entire CT scanning couch (all couch heights and sizes) to obtain a final calibration table with 2,017,920 matches (Supplementary Data Sharing).

### Independent Multicohort Testing

**Figure 4:**
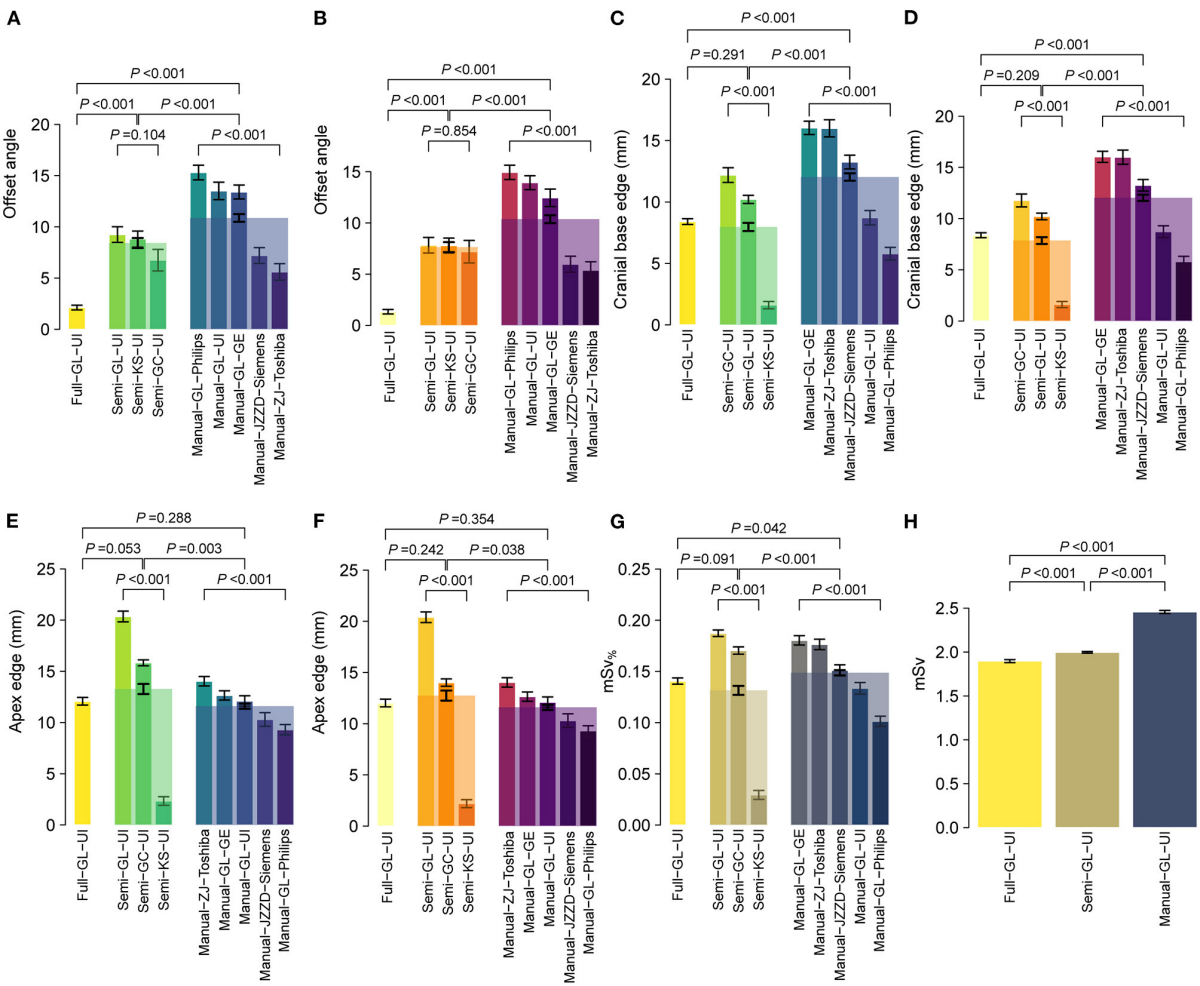
Performance of CAPITAL-CT under three clinical scenarios regarding multiple aspects, including **(A,B)** offset angle, **(C,D)** apex, and **(E,F)** cranial base edge, **(G)** additional *mSv*_%_, and **(H)** absolute *mSv*_%_. Bars here present the mean ± standard error of the mean (SEM), and bars with transparent colors were generated by pooling data from multiple hospitals under specific scenarios. Statistical *P*-values were calculated by a two-sample Student's *t*-test or one-way ANOVA for multiple group comparisons.

Three scenarios were adopted for independent multicohort testing: full navigation by CAPITAL-CT, semi-navigation by CAPITAL-CT and complete manual operation. To further explore the clinical practicability of CAPITAL-CT, we enrolled a total of 1,124 patients. Six features were measured, including offset angle, apex edge, cranial base edge, the distance from both lateral ventricles to the intracranial plate, and the distance between bilateral ventricles. Using BA analyses, we reasoned that the scanning results between the two radiologists were highly consistent regarding these features (all slope tests *P* > 0.05, Figure 4), except for the distance from the left ventricle to the intracranial plate under the semi-navigation scenario that presented significant differences (slope test *P* = 0.014) between the two radiologists (Supplementary Figure 5). Although the scanning offset angle under specific manual scenarios varied greatly among hospitals (all *P* < 0.001; Supplementary Table 7), when we pooled the results of specific scenarios, we found that the full navigation scenario had a significantly lower scanning offset angle than others (both *P* < 0.001) for both radiologists. Likewise, all semi-navigation scenarios outperformed manual scenarios regarding offset angle (both *P* < 0.001). Additionally, the scanning length of the cranial base when fully navigated or assisted by CAPITAL-CT was significantly lower than that achieved in the manual scenario (both *P* < 0.001), whereas full navigation and semi-navigation scenarios were comparable (both *P* > 0.2). However, considering the scan of the skull roof, manual scenario performance was comparable to full scenario performance (both *P* > 0.2), whereas the semiautomatic mode of man-machine coupling tended to have extra scanning length, which was most susceptible to radiographers' distrust of AI. We further calculated the absolute effective dose *mSv* per patient and computed an additional dose proportion, namely, additional *mSv*_%_, to eliminate the baseline effect of different scanning protocols. Similar to the offset angle, additional *mSv*_%_ varied significantly among hospitals under specific semi-navigation and manual scenarios (both *P* < 0.001); as expected, manual scanning had the highest effective dose compared to that of the other scenarios (both *P* < 0.05). Since different scanning protocols were used in different manufactories, *mSv* was compared within three scenarios considering United Imaging Healthcare scanners only. In this context, the measurement of *mSv* gradually increased in the full navigation, seminavigation and manual scenarios, in which the full scenario showed the lowest *mSv* and the manual scenario showed the highest (all *P* < 0.001).

### Reproducibility During Reexamination

**Figure 5:**
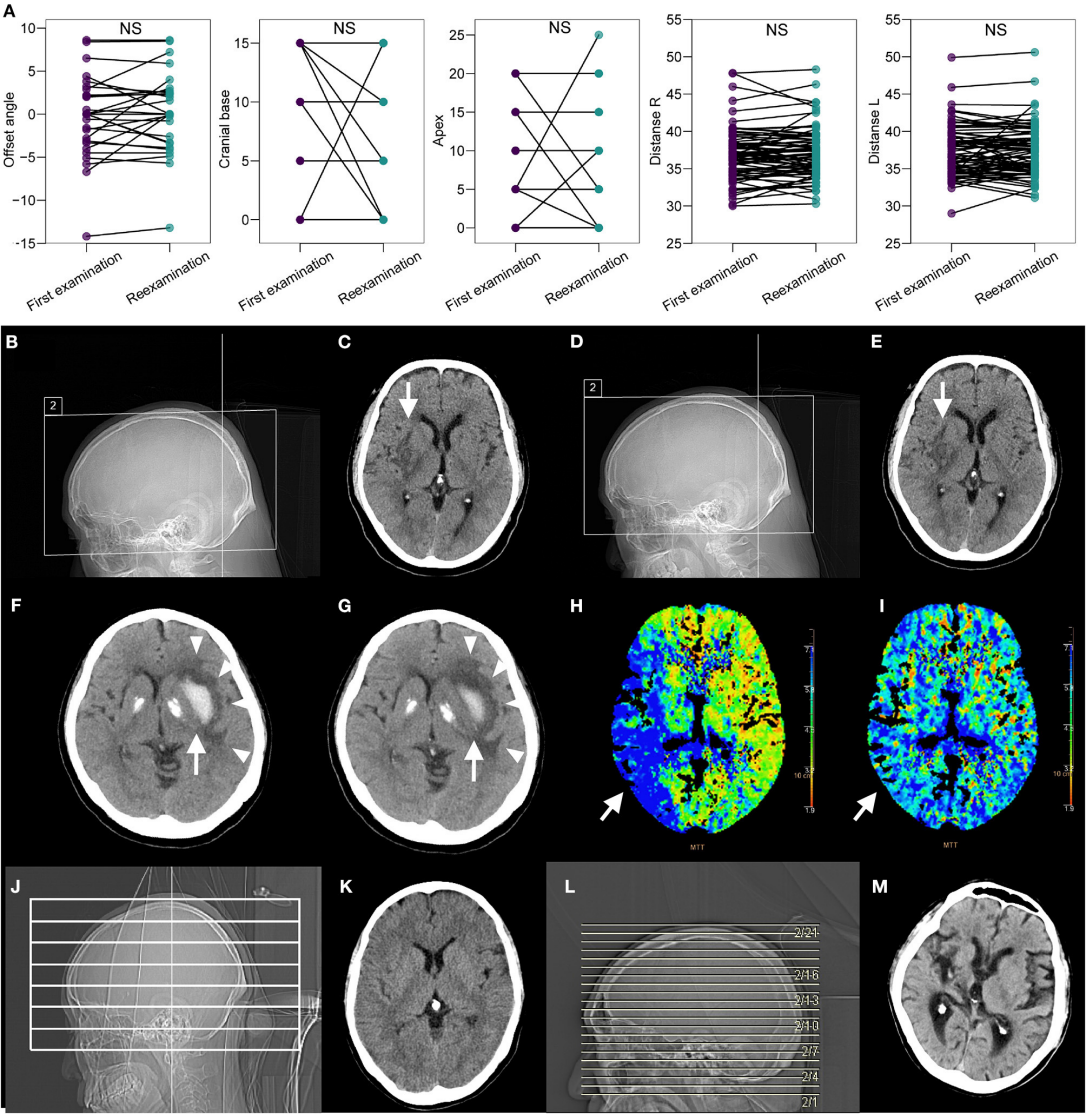
Repeatability of CAPITAL-CT compared with the repeatability of traditional CT. **(A)** Pairwise comparison revealed no significant difference between the first examination session and the reexamination session regarding offset angle, apex edge, cranial base edge, distance from both lateral ventricles to the intracranial plate, and the distance between bilateral ventricles (all paired *t*-test *P* > 0.05 and labels as ns for non-significance) for images collected from a total of 77 patients who required reexamination. **(B–E)** The patient diagnosed with ischemic cerebral infarction in the right basal ganglia (white arrow) has an ASPECTS score of 7 points. The score was reassessed as 7 points upon reexamination because the images obtained from the first examination and reexamination almost completely overlapped. **(F,G)** In the patient with cerebral infarction in the area dominated by the right middle cerebral artery (white arrow), the comparison of the mean transit time (MTT) images before and after thrombus recanalization showed that the levels were consistent, and the MTT before and after the thrombus had obvious improvement in blood flow. **(H,I)** For the patient with hemorrhagic stroke in the left basal ganglia area, the white hematoma (white arrow) was found to be significantly reduced, but the surrounding edema (white triangles) did not change after a few days of reexamination, and the images of the two sequences completely overlapped. **(J–M)** Compared with other traditional CT imaging results, CAPITAL-CT-derived images are highly reproducible.

For patients who required subsequent examinations (*n* = 77), images from both the first and second examinations were obtained and reviewed. There was no significant difference between the first and reexaminations regarding offset angle, apex edge, cranial base edge, the distance from both lateral ventricles to the intracranial plate, or the distance between bilateral ventricles (all paired *t*-test *P* > 0.05; Figure 5A). To visually display the difference in image acquisition and quality under two extreme scenarios (i.e., full navigation and manual operation), we selected three cases diagnosed with stroke (two of ischemic stroke and one of hemorrhagic stroke) that required long-term CT follow-up. Specifically, the positioning box of cranial base scanning was more accurate on topograms under the full navigation scenario; such topograms/slices could be compared with first-examination images during re-examination (Figures 5B–E) whereas the images varied greatly under the manual operation scenario, which was also reflected in the quality of transverse images. In two cases of ischemic stroke, slices were reproducible and comparable intuitively whether using non-enhanced or enhanced CT/CTP (Figures 5C–G). In the case of hemorrhagic stroke, the relationship between the hemorrhagic lesion and surrounding edema could be perfectly compared in parallel during the first and second examinations (Figures 5H,I), which might be impossible for images obtained manually in traditional methods (Figures 5J–M).

## Discussion

It is delightful to witness that AI has permeated many clinical scenarios, mainly focusing on pulmonary nodules, rib fractures and some special forms of pneumonia (20–24). However, some researchers highlighted the sizeable gap that still exists in the most upstream of medical image acquisition (25). We herein targeted stroke, combined AI with medical images and developed a clinically applicable scanning approach, which filled this gap to some extent.

The imaging process of CAPITAL-CT basically simulated and followed the clinical technician's decisions and operations (Supplementary Video 1). In terms of specific network details, we adopted relatively stable networks. First, the RPN was chosen as the core network for facial boundary detection, which successfully recognized human faces and further effectively located the scope of the human skull with remarkedly high accuracy. As shown in Supplementary Video 1, whether male or female patients wore masks or even moved their bodies significantly, our network could achieve real-time detection, which signifies its capability of accurately locating the skull of the patient. In addition, we applied V-NET, which has more advanced 3-dimensional and multioutput functions than U-NET, to identify the segmentation process based on the topograms (16, 17), which determined the location of the OML by segmenting the approximate triangle of the orbit and the external auditory meatus. Additionally, we detected and segmented the area of the brain so that we could determine the range of axial or helical scanning. Since the subjects observed by the camera would follow the law of the “perspective effect”, we continued an efficient calibration strategy according to our previous study (18). In clinical setting, we found that it takes about 4–5 min for a skull scan with traditional CT, while the scanning process completely guided by CAPITAL-CT only takes about 1/3 to half the time consumed by the traditional method because automated scanning without human interference greatly simplifies the scanning process (Supplementary Video 1). We summarized some aspects of CAPITAL-CT and compared them to other technologies in Supplementary Table 8.

Compared to the traditional scanning approach, CAPITAL-assisted scanning performed exceptionally well when using fewer imaging angles, providing repeatable images on the same level, created fewer invalid images, and revealed more symmetrical ventricles. The imaging angle is essential during scanning since it directly affects the reproducibility of patient images at the same level, which further affects the direct observation and judgment of intracranial lesions by radiologists and other clinicians. Reproducible slice images within the same level are directly favorable for the following four medical aspects: (i) avoiding variation in the Alberta stroke program early CT score (ASPECTS), which favors early assessment of ischemic changes (26); (ii) acquiring CTP and non-contrast CT images to assess the ischemic penumbra of stroke patients (27, 28); (iii) extracting radiomics information from ROIs in cranial images (29, 30); and (iv) affecting the influence of quality control in radiologic images (31, 32). In addition, it is of interest that lower-ranking hospitals seemed to have better performance in cranial symmetry than higher-ranking hospitals, which might be related to the lower reception load in lower-ranking hospitals, leading to lower working intensity and more delicate operations by radiologists.

Clinically, simple, accurate and systematic non-contrast CT (non-contrast CT, NCCT) imaging is widely harnessed to convey the condition of patients with acute ischemic stroke; such imaging plays an important role in the treatment decision-making by neurologists (33). However, neurologists observe a small area of early ischemic change (EIC) based on the NCCT images of the skull, which has two characteristics: (i) a strong time dependence and (ii) a low consistency between pre- and post-NCCT examination (34), Notably, for important neural pathways, nuclei (e.g., small ischemic changes in caudate nucleus, lentiform nucleus, or inner capsule) require rapid and stable evaluation from clinicians to propose appropriate treatment. For patients with hemorrhagic stroke, neurosurgeons pay more attention to changes in the lesions of functional and non-functional brain tissues over time, as well as changes in the size and shape of the lesions. If there is inconsistency caused by human subjective factors in the morphology of bleeding during the pre- and post-NCCT examination, a serious impact will pose on clinical decision-making. Therefore, such standardized and repeatable CT examinations provide by CAPITAL-CT will benefit both neurologist and neurosurgeon in clinical setting.

Limitations should be acknowledged, however. First, all participants were Chinese, and no other races were enrolled. Second, patients who could only be in the lateral, prone and other special conditions were not included due to their extreme rarity in clinical practice. Third, pediatric patients as well as unconscious patients were not enrolled because they could hardly control their behavior. Fourth, CAPITAL-CT currently supports scanners that are produced by United Imaging; however, the cost to install our device on other manufacturers' machines is very low. Another important concern is potential privacy issues during facial detection, and we do protect the patients' privacy and we would like to declare here. First, facial detection is a real-time capture performed by CAPTICAL-CT and will not be stored in either local machine or cloud. Second, the facial detection model of RPN was trained on public database (i.e., WIDER FACE) with 12,862 images, including 76,382 human faces with the provided position as the gold standard. Since the training data is publicly available and the training data is used for academic purpose, no invasion of privacy existed in this study.

In summary, the development of CAPITAL-CT could simplify the work of radiologists; individual images obtained by CAPITAL-CT are standard and reproducible, which is of great help in the follow-up of clinical stroke conditions and multifield research in neuroscience.

## Data Availability Statement

The datasets presented in this article are not readily available because due to the importance of protecting patient privacy, all data involved in the testing set for facial detection are not accessible and will not be stored on local servers for long periods of time. Requests to access the datasets should be directed to https://pan.baidu.com/s/1gyNu-XC6OuPT5UF7xASEbQ [Code: ZBZX].

## Ethics Statement

The studies involving human participants were reviewed and approved by Nanjing Drum Tower Hospital, Gaochun Dongba Central Hospital, Kunshan People's Hospital, Jing Ling Hospital, The Eighth Affiliated Hospital of Sun Yat-sen University, Zhanjiang Hospital Affiliated to Guangdong Medical University. Written informed consent for participation was not required for this study in accordance with the national legislation and the institutional requirements. Written informed consent was not obtained from the individual(s) for the publication of any potentially identifiable images or data included in this article.

## Author Contributions

YW, XL, JZha, and JZhu: conceptualization. YW, XL, JZha, JZhu, WL, XZhan, ML, TC, MY, and XF: experimental and data studies. RH, XM, SD, HH, XZhao, WH, CL, and XW: technical support and construction of artificial intelligence network. XL, JZhu, LJ, and FY: statistical analysis. YW, XL, JZha, JZhu, and FY: manuscript editing. YW and FY: funding acquistion. XL and FY: supervision. All authors contributed to the article and approved the submitted version.

## Funding

This work was supported by the Active Components of Natural Medicines and Independent Research Projects of the State Key Laboratory in 2020 (SKLNMZZ202016), the National Key R&D Program of China (2019YFC1711000), the Key R&D Program of Jiangsu Province [Social Development] (BE2020694), the China Postdoctoral Science Foundation (2019M651805), the National Natural Science Foundation of China (81973145), China Postdoctoral Science Foundation Special Fund for Pneumonia Epidemic Prevention and Control (Grant No. 212977), and Nantong Science and Technology Project [2021 Social and People's Livelihood Project-Public Health] (Grant No. MS12021102).

## Conflict of Interest

XM, RH, SD, HH, XZhao, XW, and WH were employed by the company Shanghai United Imaging Healthcare Co., Ltd. The remaining authors declare that the research was conducted in the absence of any commercial or financial relationships that could be construed as a potential conflict of interest.

## Publisher's Note

All claims expressed in this article are solely those of the authors and do not necessarily represent those of their affiliated organizations, or those of the publisher, the editors and the reviewers. Any product that may be evaluated in this article, or claim that may be made by its manufacturer, is not guaranteed or endorsed by the publisher.

## Abbreviations

CT: computed tomography
AI: artificial intelligence
CAD: computer-aided design
CAPITAL: Cranial Automatic Planbox Imaging Towards AmeLiorating neuroscience
ROI: regions of interest
OML: orbitomeatal baseline
SML: supraorbitomeatal line
RBL: Reid's baseline
DLP: dose length product
SEM: standard error of the mean
BA: Bland-Altman.
