# Corrigendum: Deep Learning-Enabled Clinically Applicable CT Planbox for Stroke With High Accuracy and Repeatability

**DOI:** 10.3389/fneur.2022.921992

**Published:** 2022-05-09

**Authors:** Yang Wang, Junkai Zhu, Jinli Zhao, Wenyi Li, Xin Zhang, Xiaolin Meng, Taige Chen, Ming Li, Meiping Ye, Renfang Hu, Shidan Dou, Huayin Hao, Xiaofen Zhao, Xiaoming Wu, Wei Hu, Cheng Li, Xiaole Fan, Liyun Jiang, Xiaofan Lu, Fangrong Yan

**Affiliations:** ^1^Department of Radiology, Zhujiang Hospital, Southern Medical University, Guangzhou, China; ^2^State Key Laboratory of Natural Medicines, Research Center of Biostatistics and Computational Pharmacy, China Pharmaceutical University, Nanjing, China; ^3^Department of Radiology, The Affiliated Hospital of Nantong University, Nantong, China; ^4^Department of Endocrinology, Tongren Hospital Affiliated to Shanghai Jiao Tong University School of Medicine, Shanghai, China; ^5^Department of Neurology, Drum Tower Hospital, Medical School and The State Key Laboratory of Pharmaceutical Biotechnology, Institute of Brain Science, Nanjing University, Nanjing, China; ^6^Research & Advanced Algorithm Department of HSW BU, Shanghai United Imaging Healthcare Co., Ltd., Shanghai, China; ^7^Medical School of Nanjing University, Nanjing, China; ^8^Calibration Physical Algorithm Department of CT BU, Shanghai United Imaging Healthcare Co., Ltd., Shanghai, China; ^9^Clinical Workflow and Clinical Verification Department of CT BU, Shanghai United Imaging Healthcare Co., Ltd., Shanghai, China; ^10^Department of CT BU, Shanghai United Imaging Healthcare Co., Ltd., Shanghai, China; ^11^Department of Radiology, The Second Affiliated Hospital of Nantong University, Nantong, China

**Keywords:** stroke, deep learning, computed tomography, automatic cranial scanning, accurate and repeatable images

In the published article, there was an error in affiliation “1.” Instead of “Department of Radiology, The Affiliated Nanjing Drum Tower Hospital of Nanjing University Medical School, Nanjing, China,” it should be “Department of Radiology, Zhujiang Hospital, Southern Medical University, Guangzhou, China.”

The authors apologize for this error and state that this does not change the scientific conclusions of the article in any way. The original article has been updated.

## Publisher's Note

All claims expressed in this article are solely those of the authors and do not necessarily represent those of their affiliated organizations, or those of the publisher, the editors and the reviewers. Any product that may be evaluated in this article, or claim that may be made by its manufacturer, is not guaranteed or endorsed by the publisher.

