# Supplementary material for: Deep Learning-Enabled Clinically Applicable CT Planbox for Stroke With High Accuracy and Repeatability
Source: Front Neurol. 2022 Mar 11;13:755492. doi: 10.3389/fneur.2022.755492 (PMC8961979; doi:10.3389/fneur.2022.755492)
Supplement: Supplementary file 1 [file Table_1.DOCX]

**DEEP LEARNING-ENABLED CLINICALLY APPLICABLE CT PLANBOX FOR STROKE WITH HIGH ACCURACY AND REPEATABILITY**

**INDEX OF SUPPLEMENTARY MATERIALS**

**SUPPLEMENTARY MATERIALS AND METHODS. . . . . . . .. Page 2**

**SUPPLEMENTARY TABLES S1 – 8. . . . . . . . . . . . . . . . . . . . . . . Page 8**

**SUPPLEMENTARY FIGURES S1 – 5 . . . . . . . . . . . . . . . . . . . . . Page 14**

**SUPPLEMENTARY MATERIALS AND METHODS**

**RPN and VPN development**

The region proposal network (RPN) was used for facial detection and to specify the jaw’s outer edge as the initial point, colored red, for cranial topogram scans. Specifically, the final model was selected after being trained through the public database (i.e., WIDER FACE) with 12,862 images, including 76,382 human faces with the provided position as the gold standard. A camera, which was assembled, placed on the ceiling and connected to the United Imaging Healthcare CT device, collected a total of 500 images that were used to test the final model in which the gold standard was labeled by two radiologists. The RPN was trained with 170,000 epochs on the training set, and a smooth L1 was determined by the loss of border regression for performance evaluation as below:

$${smooth}_{L_{1}}\left( x \right)=\left\{ \begin{aligned} {0.5x}^{2}, if \left| x \right|<1 \\ \left| x \right|-0.5, otherwise \end{aligned} \right.$$

V-Net was harnessed to segment the two ROIs on topograms because we reasoned that, compared with U-NET, V-NET has 3-dimensional and multioutput functions. Specifically, the cranial topogram dataset obtained between June 2016 and January 2018 included 295 topograms that served as training data and 45 topograms that served as testing data. Two ROIs in the topograms were labeled by two radiologists as the gold standard. The source code of V-Net was modified to achieve multilabel segmentation on 2-dimensional data according to our previous study. Data were preprocessed and normalized with a uniform size of 512 × 512 during the training process, in which the normalized data were further input into V-Net for end-to-end training with 422,499 iterations. The Dice coefficient was calculated as a loss function for performance evaluation as below:

$$Dice^{'}s coefficient=\frac{2\sum_{i}^{N} p_{i}g_{i}}{\sum_{i}^{N} p_{i}^{2}+\sum_{i}^{N} g_{i}^{2}}$$

The total number of pixels that needed to be traversed was denoted as *N*, the predicted segmentation region was defined as $\left\{ p_{i}\in P \right\}$, and the gold standard segmentation region was designates as $\left\{ g_{i}\in G \right\}$.

**Principle of scanning couch displacement and camera calibration**

**Scanning couch displacement process**

The vertical and horizontal directions can be simply used to describe the displacement of the scanning couch. 1) Vertical direction: Since it is difficult for 2D cameras to obtain the subject’s thickness, the national standard for human dimensions of Chinese adults (GB10000-88, untranslated, see more details in the Data Sharing section) has a set of default organ thicknesses and default couch heights under different scanning protocols. Therefore, the scanning couch automatically moves vertically to the corresponding height according to the specific scanning protocol (e.g., cranial imaging with a default couch height of 820 mm (black dashed line) and compensatory height of 190 mm (mattress and body thickness)). 2) Horizontal direction: There is no need for redundant components in this process (e.g., the standard chessboard ^1^), but the sparse sampling method is involved here as an innovative application, which is essentially a simple calibration method for pattern recognition (Figure S1).

**Camera calibration process**

In general, there are 6 steps in the camera debugging process: 1) Before the calibration process, the camera should be properly installed. After that, a white bar is used as the recognition object and must be clearly visible in the field of vision (FOV) of the camera. 2) The couch moves to the scan center until the white bar is aligned with the inner laser line (isocenter). Then, the couch code (horizontal couch position) of isocenter *C_0_* should be recorded. 3) Under different couch heights (vertical couch position, h), the white bar is moved in the range of the couch code (horizontal couch position, c). As the couch traverses each position, the camera takes one photo, records the corresponding position and saves the image as the couch traverses each position. 4) The horizontal center pixel of the white bar can be found in a set of couch positions in the recognition process (Figure S2-3). The mapping relation of the white bar center can be transformed from real physical space to camera pixel space via the following formula *P* = *f* (*ℎ*, *c*) based on the marked horizontal pixels *p* corresponding to a specific couch position [*ℎ*, *c*]. 5) A mapping relation function is identified to calculate the marked horizontal pixels *P* corresponding to all couch heights H and couch codes C, which are inferred by model fitting (linear interpolation), interpolation and extrapolation: *P* = *f* (*H*, *C*). 6) The final calibration table is generated as [*H*, *P*, *L*], where the horizontal move length *L* is denoted by *C* minus *C_0_*. Specifically, three essential steps 3-5 and several issues are described in detail below.

***a) Camera calibration:*** The CT scanning couch from the original manufacturer has three inherent white symbols for the installation of CT hardware: 1) a light white ‘inner cross’ on the couch side closest to the scanning gantry, 2) an ‘outer cross’ on the tail of the couch away from the gantry, and 3) a regular and obvious ‘white bar’ next to the ‘outer cross’ (Figure S2). Only the ‘white bar’ can be displaced to the boundary area close to the visual field within the camera when the scanning couch moves in the horizontal direction. For this reason, when capturing images at high altitude, such installation of the camera and the photography requirements can enlarge the view range and enhance the anti-interference ability of the camera. Therefore, the ‘white bar’ is selected as the recognition target in the camera calibration process, which requires photos of the white bar that are located at different positions on the CT scanning couch.

Generally, the visual law of the ‘perspective effect’ is well known, and it is no exception in the imaging process. This law notes that the dynamic bias in the imaging process is an inevitable problem and needs to be solved, especially for the recognition of white bars (Figure S3). We solved this problem by using the camera to collect pictures of fixed markers at different spatial positions, locating pixel positions of the markers within the camera’s visual field via the image recognition method and finally obtaining all mapping relations through numerical calculation based on the partial physical spatial position and corresponding pixel positions (Figure S3). In addition, using sparse sampling could also overcome the challenge of the blind area in the visual field of the camera (Figure S4).

Effective camera installation was determined based on the technical parameters of the camera and actual tests. We set the couch position at different couch heights/codes for taking pictures, but this setting was limited by the range of couch motion and installation range of the camera. Affected by the ‘perspective effect’, the camera took several pictures of the white bars at different couch heights/codes (corresponding to the red dot in Figure S4). However, the white bar was blocked by the gantry on the left side of the red dot when the white bar was outside the visual field of the camera on the right side. In addition, the scanning couch could only move vertically to avoid a horizontal collision in the area below; therefore, we set up various couch code positions at multiple higher couch sites.

In particular, it was inevitable that the reflection of the illumination source on the scanning couch would cause spot interference during camera photography (Figure S3). Due to the reflective black glossy material, the spot overlapped with the white bar and was captured by the camera, making it unrecognizable. We tried to adjust the lights and turn off all general lighting by just using the camera's night vision mode. However, the fill light of the camera also caused interference with the white bar in night vision mode. Therefore, we eventually adjust the installation position of the camera to avoid this issue.

***b) White bar recognition:*** The white bar is the inherent mark of the CT scanning couch with a concise shape, which is conducive to recognition and calibration. Therefore, we adopted the classic ‘template matching method’ to perform the image recognition procedure through a standard pattern of the white bar as a template to match all the collected images, to identify the white bar in various distorted statuses and to calculate the location of the pixel center ^2^. In the testing phase, the camera enables precise recognition of all the white bars (Figure S3), even in night vision mode, which interferes with fill light. The large red box indicated that the camera roughly screened the approximate region of interest (ROI) first. The small red rectangle is the more accurate white bar ROI recognized by the camera. The red cross is marked for identification of the position of the center pixel of the white bar (Figure S3). In addition to light interference, the geometric distortion of the image captured by the camera also affects the recognition process, i.e., different scanning couch positions in the visual field of the camera determine different shapes and sizes of the white bar. For example, the image presented is relatively large when the white bar is at a higher position of the scanning couch; the original rectangular shape is distorted and, of course, more extreme positions also exist when the white bar is farther away from the camera (Figure S3). To address this particular issue, we used an image recognition method that is robust to geometric distortion because of the 100% white bar recognition rate (see more details in the Data Sharing section).

***c) Generating a calibration table:*** Due to the use of the overall layout of the 2D camera on the scanning couch, we could only observe the movement and trajectory of the subject in the horizontal direction in the visual field of the camera, whereas the physical height (thickness) information for the longitudinal direction failed to be captured. For this reason, we stipulated that the motion of the scanning couch (the line connecting the two crosses on the scanning couch) must be parallel to the lateral edge of the visual field of the camera, which means that the horizontal shape pixel information of the white bar is the only requirement.

The overall process for generating the calibration table is shown in Figure S4. According to the collected couch height and the corresponding white horizontal center pixel (the reddish area in Figure S4a-b) under various couch codes, the sparse sampling method was needed to fit, interpolate and extrapolate to obtain the horizontal pixel center value of the white bars for the entire couch height/code (red in Figure S4c-d and the surrounding blue area). This method included the following key steps:

1) In the position of the scanning couch (the light red area in Figure S4c), we assumed that the horizontal pixel position *ρ* of the center of the white bar was linear with the couch height, ℎ: *ρ* = *k* × ℎ + *b*, (Figure S4d) under the same couch code *c*.

2) A series of pixel positions *ρ* under couch code *c* could be derived by the formula — *P* = *f* (ℎ, *c*) by considering all couch heights in the equation above, including those that failed to be collected at lower couch heights ℎ (small red dots, Figure S4c).

3) The couch code *C* of all pixel ranges *P* was obtained by cubic spline interpolation using the already obtained couch code and pixel relationship through the formula — *P* = *f* (ℎ, *c*) at the same couch height *H*.

4) Finally, the calibration table could be stored as a correction table in the format required by the regulations. The absolute amount (the horizontal moving distance of the scanning couch) is required if the camera-assisted automatic positioning function was used. Therefore, there was a need for us to find the couch code when the white bar was scanning the target position and to record the couch code *C_0_* when the inner laser light was aligned with the white bar. The corresponding horizontal couch distance was *L* = *C* − *C_0_*. The format of the record was: [*H*, *P*, *L*].

**The usage of the calibration table**

It is worth noting that due to the lack of the subject height or thickness information and the interference of the imaging law, it was still insufficient to directly use the currently generated calibration table of white bars at different couch heights. In this context, we implicitly took the national standard for human dimensions of Chinese adults (GB10000-88) and set the compensatory height to the default couch height under specific scanning protocols. This amendment to the calibration table to simultaneously minimize the hardware cost and further improved the feasibility of translational medicine. Briefly, this calibration process provided a mapping table of different couch heights; the 2D camera failed to obtain the thickness information (height or depth) of the subject when the subject lay on the scanning couch. Moreover, would encounter two issues if this calibration table were used directly because of the existence of imaging effects. 1) The couch height of the lateral position of the subject at the scanning center, which is equal to the inaccurate center position of the lateral position of the topogram, is unknown. 2) The imaging effect and geometric distortion in the horizontal direction may influence the horizontal positioning accuracy. Considering the hardware cost, we included the default couch height under different scanning protocols and the organ thickness in the calibration table (GB10000-88).

The radiographer has indicated that he or she wants to move the position to the isocenter when the camera automatically recognizes a specific position on the screen or when the radiographer manually clicks on a specific position on the screen showing the subject. At this moment, the system first acquires the current scanning protocol to derive the default couch height at the starting position as well as the compensatory height that needs to be included in the calibration table; then, the scanning couch height/code and the horizontal pixel are obtained. According to the cumulative height and the horizontal pixel, the calibration table can be used to map the unique horizontal moving distance. Subsequently, we can extract the horizontal moving distance from the current couch code and finally identify the target couch code. A manual operation process is still needed in this phase. After confirmation by the radiographer, the scanning couch moves vertically to the default couch height under the scanning protocol and then moves horizontally to the target couch code for this scan.

**Accuracy testing of the camera - CT couch positioning**

The inherent marks are used, e.g., the inner/outer cross and the obvious marker (vertical line), to record the so-called couch code ‘gold standard’ when moving to the isocenter. After that, the radiographer manually clicks on the marker to automatically move the couch, records the couch code at this time, and then compares it with the gold standard to measure the positioning error and accuracy. In this experimental design, there are two sizes of scanning couches with minimum couch heights of 480 mm and 600 mm as well as maximum horizontal displacements of 1,930 mm and 2,180 mm. We set the preset error allowed in this experiment to ± 20 mm, but the error was approximately 10 mm according to the actual experimental test results (Table S4-6). The accuracy in other locations and subsequent scans can be guaranteed by the debugging results.

**SUPPLEMENTARY REFERENCES**

1. Zhang Z. A flexible new technique for camera calibration. IEEE Trans Pattern Anal Mach Intell 2000; 22: 1330-4.

2. Gonzalez RC, Woods RE. Digital image processing. Upper Saddle River, NJ: Prentice Hall; 2002.

**SUPPLEMENTARY TABLES**

**Table S1: Characteristics of objects used in this study.**

| **Parameters** | **Objects (images) metric*** |
| --- | --- |
| **Head field segmentation sets** |  |
| Number of objects (training/validation datasets) | 295/45 |
| Ratio of females to males | 1.17:1 (159:136)/0.96:1 (22:23) |
| Age (years) | 56.5 ± 1.0/61.1 ± 2.7 |
| **Validation sets** |  |
| Number of patients (independent testing datasets) | 1,124 |
| Ratio of females to male | 0.95:1 (547:577) |
| Age (years) | 52.3 ± 0.6 |
| **Different manufacturers** | **Number of patients** |
| United Imaging | 632 |
| GE | 121 |
| Philips | 114 |
| Siemens | 147 |
| Toshiba | 110 |

* Continuous values are presented as the mean ± standard error of the mean (SEM)

| **Table S2. The scanning and reconstruction parameters of CT images for independent multicohort testing** | | | | | | |
| --- | --- | --- | --- | --- | --- | --- |
| **Index** | **Sites** | | | | | |
| **Hospital names** | Nanjing Drum Tower Hospital (n=658) | Nanjing Gaochun Dongba Central Hospital (n=96) | Kunshan People's Hospital (n=113) | Nanjing Jinling Hospital (n=125) | The Eighth Affiliated Hospital of Sun Yat-sen University (n=22) | Zhanjiang Hospital Affiliated to Guangdong Medical University (n=110) |
| **CT scanners** | United Imaging uCT 780/790  GE LightSpeed VCT/Discovery 750 HD/Revolution CT  Philips Brilliance iCT | United Imaging uCT 520 | United Imaging uCT 530 | SOMATOM Emotion/Perspective CT 6/64-slices | SOMATOM Force CT 128-slices | Toshiba Aquilion ONE CT 16-slices |
| **Tube voltage (kVp)** | 120/140 | 140 | 140 | 130 | 120 | 120 |
| **Tube current (mA)** | 100 - 350 | 130 | 111 | 167 | 165 | 250 |
| **Scan options & detector** | Axial 64×1.25/80×0.5/160/128×0.65 | Helical 16 × 0.65 | Axial 16 × 0.65 | Helical 6/64 × 1.5 | Axial 128 × 0.55 | Helical 16 × 0.5 |
| **Slice thickness (mm)** | 5/10 | 5 | 5.5 | 9 | 8 | 2 |
| **Reconstruction increment** | No overlap | No overlap | No overlap | No overlap | No overlap | No overlap |
| **Convolution kernel** | H-SOFT-B/STND | H-SOFT-CA | H-SOFT-B | H30s | Hr40s/3 | FC68 |
| **Matrix** | 512×512 | 512×512 | 512×512 | 512×512 | 512×512 | 512×512 |

**Table S3. Linear association between the horizontal pixel position of white bars and couch heights at the same horizontal couch position**

| Horizontal couch positions | Coefficient | | *P* | Adjusted R^2^ |
| --- | --- | --- | --- | --- |
|  | Intercept | Couch height |  |  |
| 350 | 609.531 | -0.22 | <0.0001 | 1.00 |
| 400 | 634.031 | -0.214 | 0.0003 | 1.00 |
| 450 | 657.531 | -0.206 | 0.0003 | 1.00 |
| 500 | 678.031 | -0.194 | 0.0003 | 1.00 |
| 550 | 697.531 | -0.18 | <0.0001 | 1.00 |
| 600 | 717.531 | -0.166 | 0.0004 | 1.00 |
| 700 | 756.031 | -0.134 | 0.0007 | 1.00 |
| 900 | 841.031 | -0.074 | 0.0022 | 0.99 |
| 1100 | 918.531 | 0 | NA | NA |
| 1300 | 1003.531 | 0.066 | 0.0027 | 0.99 |
| 1500 | 1084.031 | 0.134 | 0.0007 | 1.00 |
| 1700 | 1165.031 | 0.194 | 0.0003 | 1.00 |
| 1800 | 1205.531 | 0.22 | <0.0001 | 1.00 |
| 1850 | 1224.031 | 0.234 | 0.0002 | 1.00 |
| 1900 | 1247.531 | 0.242 | 0.0005 | 1.00 |
| 1950 | 1268.031 | 0.252 | 0.0001 | 1.00 |
| 2000 | 1297.531 | 0.252 | 0.0008 | 1.00 |
| 2050 | 1318.531 | 0.26 | <0.0001 | 1.00 |
| 2100 | 1340.531 | 0.266 | 0.0002 | 1.00 |
| 2150 | 1361.031 | 0.274 | 0.0002 | 1.00 |

| \| **Table S4. Accuracy testing at Nanjing Drum Tower Hospital** \| \| \| \| \| --- \| --- \| --- \| --- \| \| Couch height (mm) \| Target couch code \| Automatic positioning couch code (mm) \| Error (mm) \| \| 480 \| 1,508.8 \| 1499.7 \| 9.1 \| \|  \| 813.7 \| 816.3 \| -2.6 \| \|  \| 78.2 \| 68.6 \| 9.6 \| \| 600 \| 1,508.8 \| 1500.5 \| 8.3 \| \|  \| 813.7 \| 821.0 \| -7.3 \| \|  \| 78.2 \| 73.1 \| 5.1 \| \| 800 \| 1508.8 \| 1503.2 \| 5.6 \| \|  \| 813.7 \| 819.7 \| -6.0 \| \|  \| 78.2 \| 88.0 \| -9.8 \| \| 950 \| 1508.8 \| 1502.8 \| 6.0 \| \|  \| 813.7 \| 816.7 \| -3.0 \| \|  \| 78.2 \| 86.6 \| -8.4 \|   **Table S5: Results of accuracy testing at Gaochun Dongba Central Hospital** | | | |
| --- | --- | --- | --- | --- | --- | --- | --- | --- | --- | --- | --- | --- | --- | --- | --- | --- | --- | --- | --- | --- | --- | --- | --- | --- | --- | --- | --- | --- | --- | --- | --- | --- | --- | --- | --- | --- | --- | --- | --- | --- | --- | --- | --- | --- | --- | --- | --- | --- | --- | --- | --- | --- | --- | --- | --- | --- | --- | --- | --- |
| Couch height (mm) | Target couch code | Automatic positioning couch code | Error |
| 600 | 1245.2 | 1,258.8 | -13.6 |
| 800 | 1245.2 | 1,47.6 | -2.4 |
| 950 | 1245.2 | 1,247.5 | -2.2 |

| **Table S6: Results of accuracy testing at Kunshan People's Hospital** | | | |
| --- | --- | --- | --- |
| Couch height (mm) | Target couch code | Automatic positioning couch code | Error |
| 480 | 1412.4 | 1418.7 | -6.3 |
| 800 | 1412.4 | 1,414.5 | -2.1 |
| 950 | 1412.4 | 1,415.6 | -3.2 |

**Table S7. Comparison of scanning efﬁciency and radiation exposure reduction among three clinical scenarios with 1,124 patients.**

|  | Clinical Scenario | | |  | *P*-value | | |
| --- | --- | --- | --- | --- | --- | --- | --- |
|  | Full (F) | Semi (S) | Manual (M) |  | F vs S | F vs M | S vs M |
| Number of patients | 166 | 349 | 609 |  |  |  |  |
| Offset angle |  |  |  |  |  |  |  |
| Radiologist 1 | 2.12 ± 0.2 | 8.42 ± 0.5 (0.104) | 10.87 ± 0.4 (<0.001) |  | <0.001 | <0.001 | <0.001 |
| Radiologist 2 | 1.33 ± 0.2 | 7.64 ± 0.5 (0.854) | 10.37 ± 0.4 (<0.001) |  | <0.001 | <0.001 | <0.001 |
| *Distance from edge (mm)* |  |  |  |  |  |  |  |
| *Apex* |  |  |  |  |  |  |  |
| Radiologist 1 | 12.08 ± 0.4 | 13.28 ± 0.5 (<0.001) | 11.60 ± 0.3 (<0.001) |  | 0.053 | 0.288 | 0.003 |
| Radiologist 2 | 12.02 ± 0.4 | 12.74 ± 0.5 (<0.001) | 11.60 ± 0.3 (<0.001) |  | 0.242 | 0.354 | 0.038 |
| *Base* |  |  |  |  |  |  |  |
| Radiologist 1 | 8.40 ± 0.2 | 7.97 ± 0.3 (<0.001) | 12.03 ± 0.3 (<0.001) |  | 0.291 | <0.001 | <0.001 |
| Radiologist 2 | 8.37 ± 0.2 | 7.86 ± 0.3 (<0.001) | 12.03 ± 0.3 (<0.001) |  | 0.209 | <0.001 | <0.001 |
| Effective dose (mSv) | 1.90 ± 0.0 | 1.75 ± 0.0 (<0.001) | 1.88 ± 0.0 (<0.001) |  | <0.001 | 0.471 | <0.001 |
| Additional dose rate (${mSv}_{\%}$) | 0.14 ± 0.0 | 0.13 ± 0.0 (<0.001) | 0.15 ± 0.0 (<0.001) |  | 0.091 | 0.042 | <0.001 |
| ^a^ Values here are presented as the mean ± standard error of the mean (SEM). | | | | | | | |
| ^b^ One-way ANOVA P-value for multiple groups comparison are presented within parenthesis. | | | | | | | |
| ^c^ Two-sample Student’s *t*-test *P*-value. | | | | | | | |

| **Table S8:** **The summarization of several aspects of CAPITAL-CT, combining artificial intelligence with cranial scanning, compared to the aspects of other technologies** | | | |
| --- | --- | --- | --- |
| Name | CAPITAL-CT scanning | Siemens scanning | GE scanning |
| Cranial scanning  (Success rate) | Yes (100%) | Yes (100%) | N/A |
| Training/testing/validation set | 76,382 images/295 patients/1,124 patients | 45 patients | N/A |
| Multicenter validation | Yes | No | No |
| Error | Boundaries of 3 mm, lines of 3° on average | 9.5 (6.8) mm | N/A |
| Reproducibility | Yes | N/A | N/A |
| Camera type | 2D | 3D | 3D |
| Commercial CT scanner | No | Yes | Yes |
| Networks or methods | RPN; V-NET; sparse sampling method | Infrared light and the time-of-flight (TOF) principle | N/A |
| Availability of source code | Yes | No | No |

**SUPPLEMENTARY FIGURES**


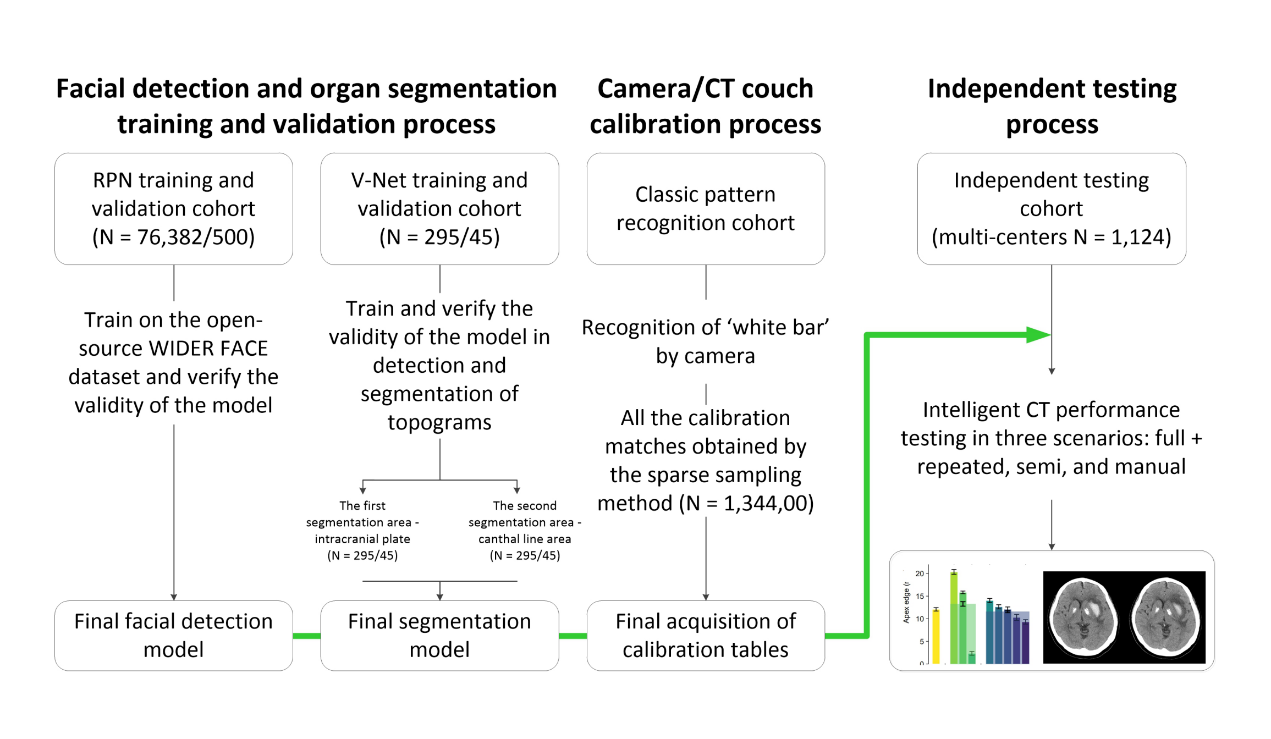


Figure S1. The entire workflow and numbers for each respective dataset. The description of three parts in this study, including model training, calibration and independent testing.


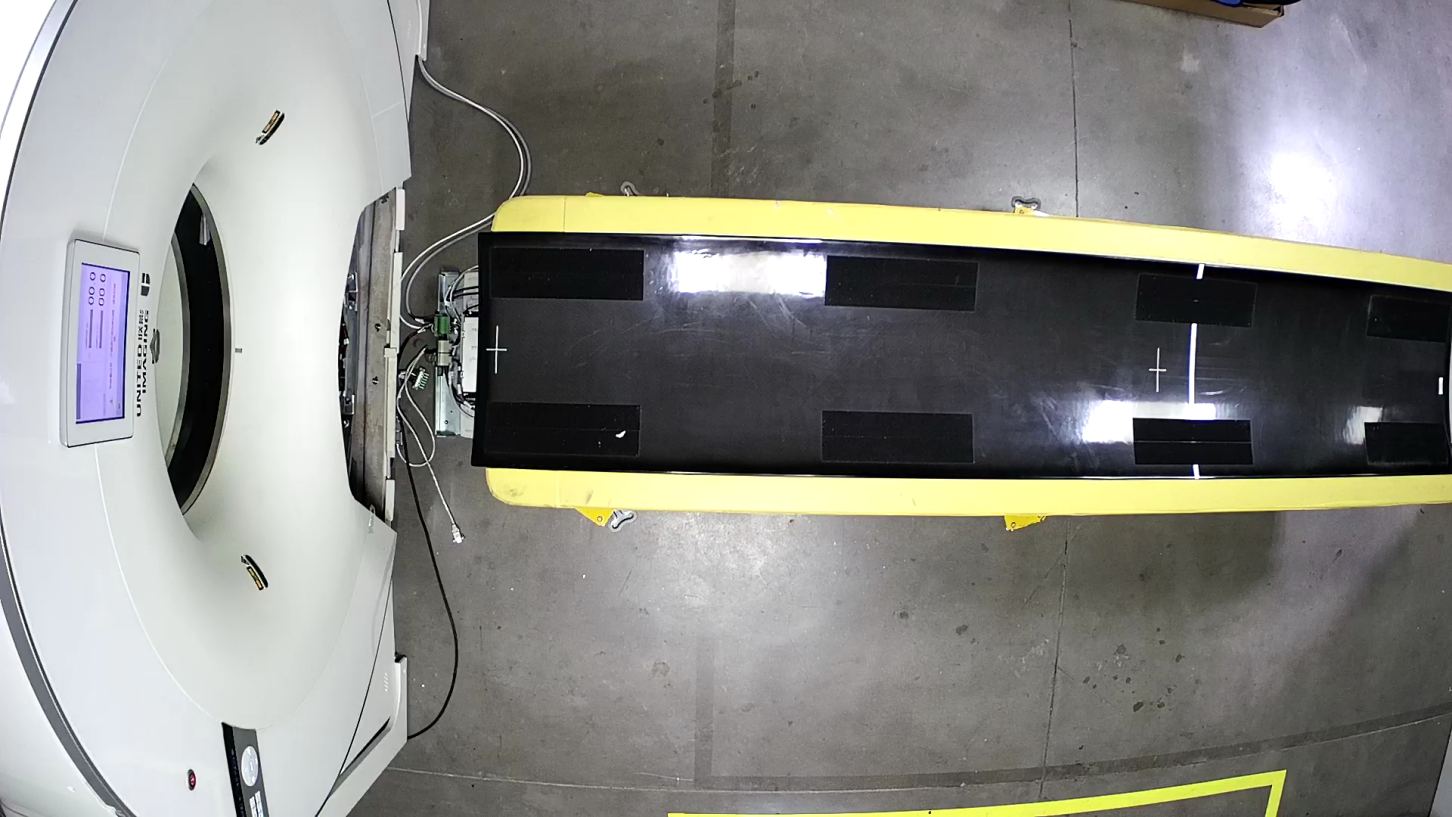


Figure S2. Symbol compositions marked on the CT scan couch. Three white symbols are printed on the black couch, namely, a pale white “inner cross” on the couch near the isocenter, a pale white “outer cross” away from the end of the couch, and an obvious “white bar” on the outer side of the couch. In addition, during the test process, the inherent markers on the scanning couch were used to test and verify the error of the camera-assisted positioning. The “inner cross” and “white bar” with obvious marks were selected as test objects.


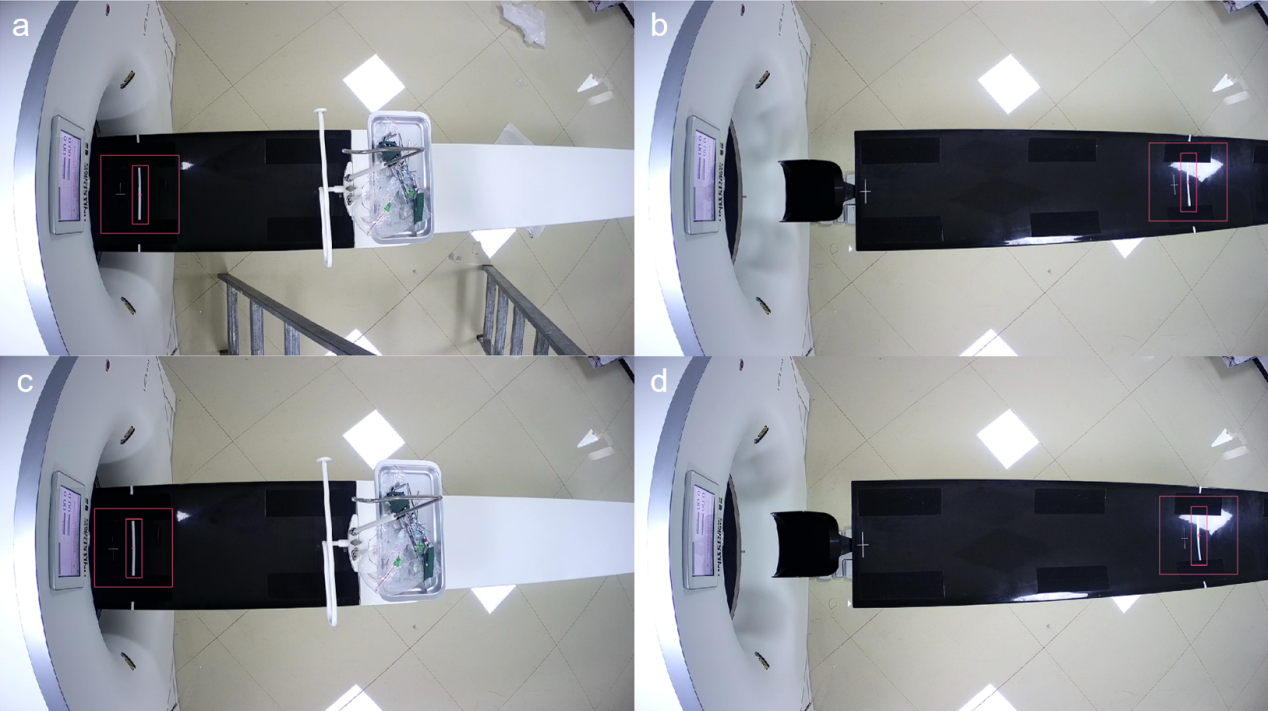


Figure S3. Four pictures in extreme positions where the scanning couch is located to test the ability of the camera to recognize the central pixel of the white bars. a) The highest and innermost position of the scanning couch and its recognition process with red boxes. b) The highest and outermost position and its recognition process with red boxes. c) The lowest and innermost position and its recognition process with red boxes. d) The lowest and outermost position and its recognition process with red boxes. Although the size and shape of the white bars have a certain degree of geometric distortion in different positions, this classic ‘template matching method’ can successfully identify and mark the exact position of the horizontal pixels in the center of the white bar with red boxes. In addition, although the reflection of the illumination source on the scanning couch caused interference, the white bar was still recognized.


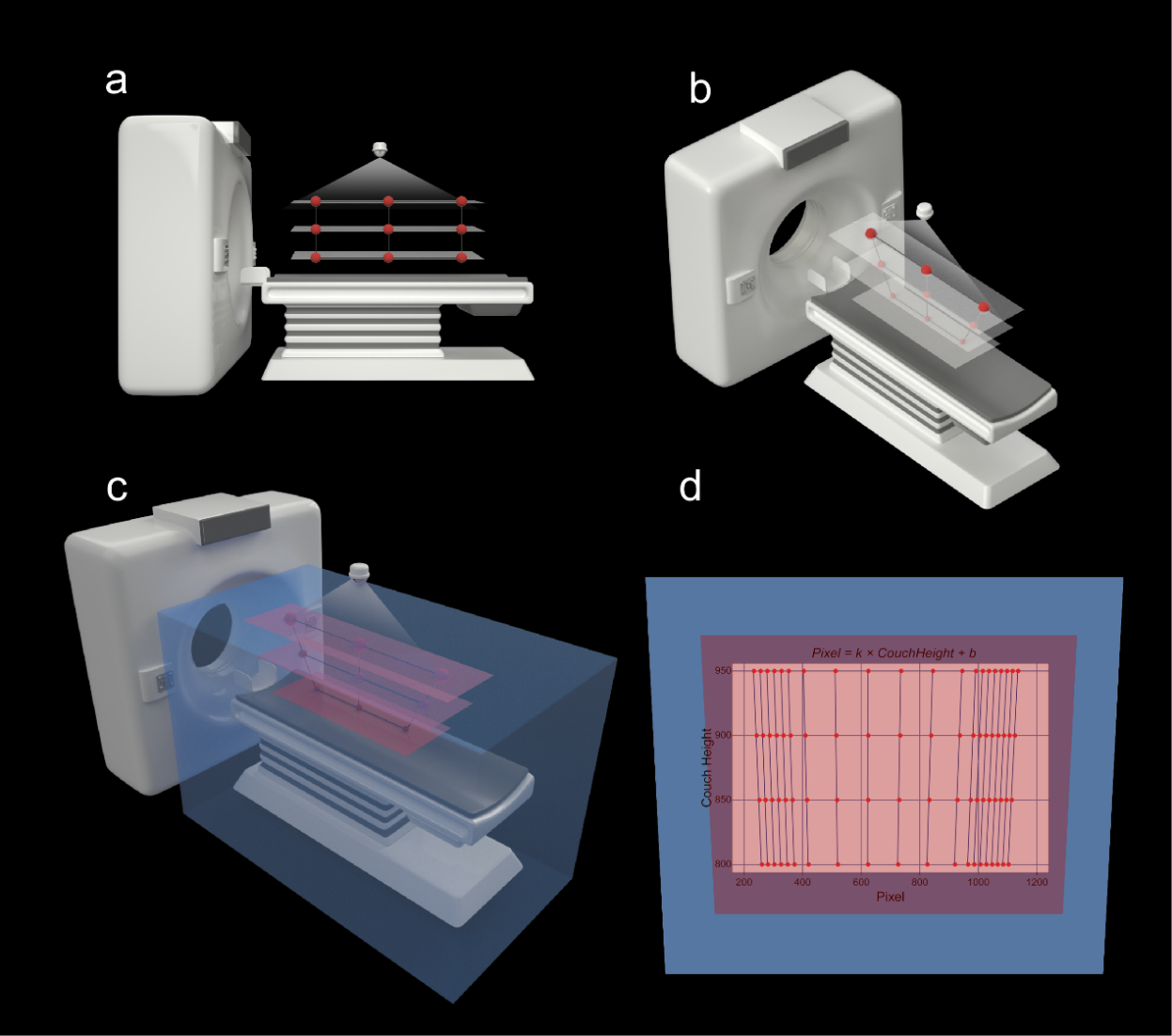


Figure S4. Schematic diagram of the subject’s imaging field in the CT scanner and data acquisition in the camera’s imaging field. In addition, the generation of a calibration table for pixel mapping between a CT scanning couch and camera imaging. a) A complete side imaging view of the CT scanner and camera; that is, when the subject shifts on the scanning couch, the size of the red dot in the center of the subject is substantially unaffected. b) The dark grey coordinate system represents the movable vertical scanning couch height and horizontal pixel code of the camera. Affected by the natural law of the perspective effect, the actual ‘white bar center point and red dot’ of the same size increases in the visual field of the camera as the couch height increases. However, due to the limitation of the camera installation position and couch movement range, the pixel position of the center of the white bar (the red dots) can be collected only at several scanning couch positions during practical operation. c) The red region represents the area that the white bar can traverse, and the surrounding blue region is unreachable but needs to be covered and calculated by the calibration table. d) Horizontal pixels (blue areas) corresponding to all continuous couch heights and couch code values are inferred based on the linear relationship between camera pixels and couch height. The red and blue areas in this image correspond to the red and blue areas in image c.


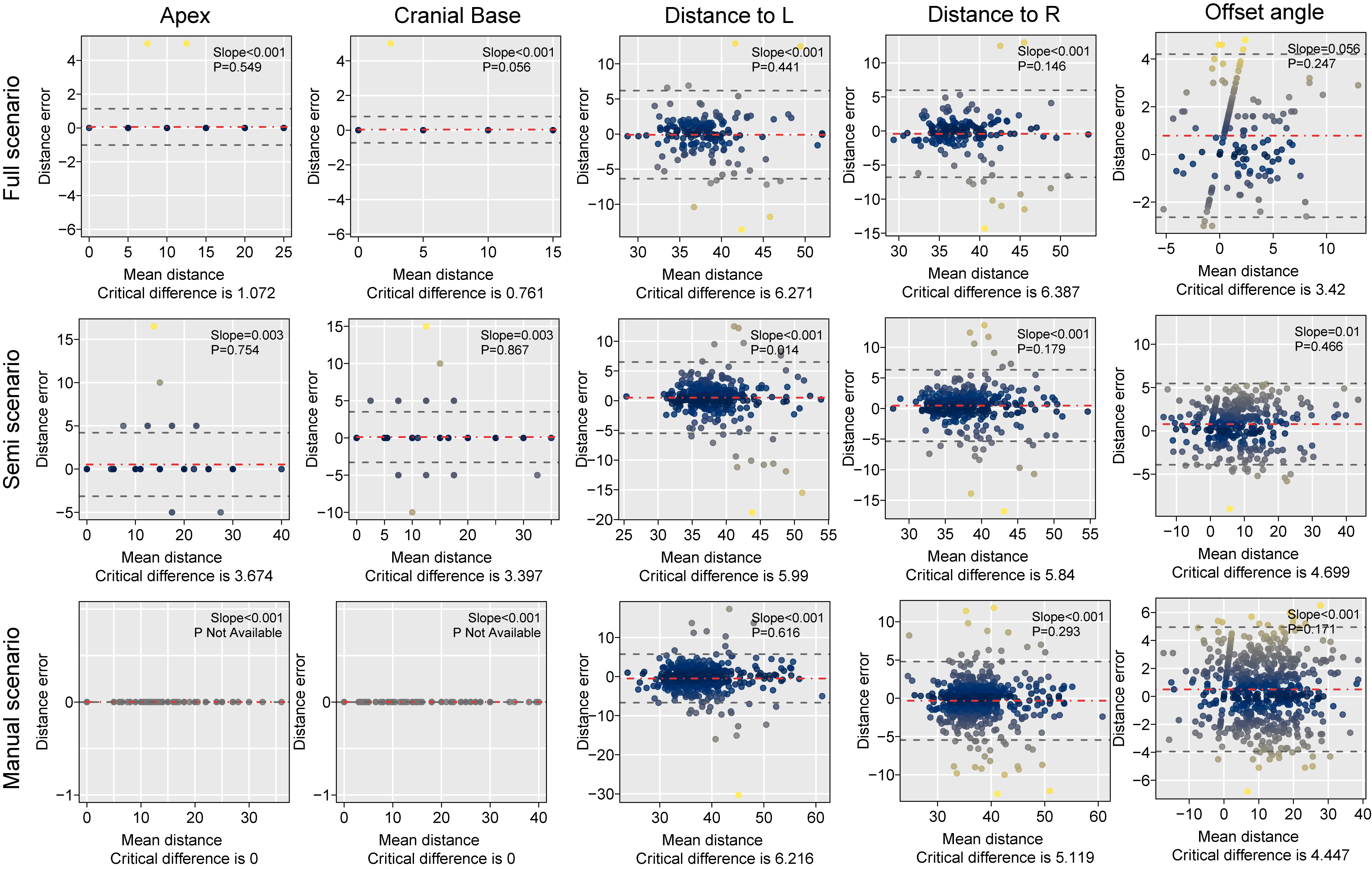


Figure S5. Bland-Altman plots reveal consistency between the two radiologists regarding five measurements in three clinical scenarios. The differences (y-axial) are plotted versus the average (x-axial) values measured by two radiologists for the cranial apex, cranial base, distance from both lateral ventricles to the intracranial plate left and right boundary, and offset angle. Slopes are calculated by linear regression of differences on average, and the corresponding statistical *P* values are provided for testing a constant difference based on a null hypothesis—the slope equals 0. The critical difference is 'two' times the standard deviation of differences and equals half the difference between the lower and upper limits.
